# Silicene Quantum Capacitance Dependent Frequency Readout to a Label-Free Detection of DNA Hybridization— A Simulation Analysis

**DOI:** 10.3390/bios11060178

**Published:** 2021-06-01

**Authors:** Md. Sazzadur Rahman, Rokaia Laizu Naima, Khatuna Jannatun Shetu, Md. Mahabub Hossain, M. Shamim Kaiser, A. S. M. Sanwar Hosen, Md. Abdul Latif Sarker, Kelvin J. A. Ooi

**Affiliations:** 1 Institute of Information Technology, Jahangirnagar University, Savar Dhaka-1342, Bangladesh; sazzad@juniv.edu; 2 Department of Electronics and Communication Engineering, Hajee Mohammad Danesh Science & Technology University, Basherhat N508, Bangladesh; rokaiyalaizu@gmail.com (R.L.N.); jannatunshetu40@gmail.com (K.J.S.); im.mahabub@gmail.com (M.M.H.); 3 Division of Computer Science and Engineering, Jeonbuk National University, Jeonju 54896, Korea; sanwar@jbnu.ac.kr; 4 Department of Electronic Engineering, Hanyang University, Seoul 04763, Korea; abdul123@hanyang.ac.kr; 5 Department of Physics, Xiamen University Malaysia, Sepang 43900, Malaysia

**Keywords:** biosensor, pH detection, quantum capacitance, resonant frequency, ISFETs

## Abstract

The use of deoxyribonucleic acid (DNA) hybridization to detect disease-related gene expression is a valuable diagnostic tool. An ion-sensitive field-effect transistor (ISFET) with a graphene layer has been utilized for detecting DNA hybridization. Silicene is a two-dimensional silicon allotrope with structural properties similar to graphene. Thus, it has recently experienced intensive scientific research interest due to its unique electrical, mechanical, and sensing characteristics. In this paper, we proposed an ISFET structure with silicene and electrolyte layers for the label-free detection of DNA hybridization. When DNA hybridization occurs, it changes the ion concentration in the surface layer of the silicene and the pH level of the electrolyte solution. The process also changes the quantum capacitance of the silicene layer and the electrical properties of the ISFET device. The quantum capacitance and the corresponding resonant frequency readout of the silicene and graphene are compared. The performance evaluation found that the changes in quantum capacitance, resonant frequency, and tuning ratio indicate that the sensitivity of silicene is much more effective than graphene.

## 1. Introduction

DNA hybridization computes the degree of similarity in pools of DNA sequences which is employed in calculating the genetic difference between two organisms. In the biological aspect, DNA hybridization provides a powerful tool that allows identification as well as cloning of specific genes, and analysis of the number of copies of the sequence in the genome.

The typical applications of hybridization studies include detecting a wide range of infectious agents, showing the occurrence of human chromosomal aberrations, detecting many genes responsible for inherited disorders, identifying hepatitis B, complex pancreatic coronary diseases, and showing how many tumors rearrange genes and amplify oncogenics [1,2,3].

DNA detection as a molecular diagnostic agent is extremely sensitive and precise. Graphene and graphene-based nanomaterials have unique properties that piqued researchers’ interest in using them to enhance the efficiency of DNA detection [3]. Since graphene was discovered and successfully applied, the 2D structures of group IV elements have become one of the most studied physical and nanoscience materials. Silicene is a silicon (Si) analogy of graphene where Si replaces C in a double honeycomb structure [4]. Recently, silicene has gained considerable attention from its theoretical and experimental point of view [5]. According to Salimian and Dideban [6], the application of a silicene nanotube-based DNA hybridization sensor in real-time analysis provides high sensitivity, high efficiency, better tunability, and lower band charge density in molecular biology. In 1994, silicene was first demonstrated as a 2D material that arises silicene as “Silic” and “ene” from graphene. Experimental evidence of silicene has opened ground breaking opportunities for theoretical and experimental physicists [7].

With the atomic resolution, silicene nano wires and silicene sheets have been grown on silver crystal as Ag (110) and Ag (111) [8]. Since its invention, it has numerous attractive physical and electrochemical properties such as high intrinsic mobility (near 1000 cm${}^{2}$/V.s) at room temperature [9], a better tunability of band gap (0.5 eV) which is necessary for field effect transistor (FET) [10,11], high speed switching, and a much stronger spin orbit coupling. The physical property of free standing silicene with a spin orbit band gap of 1.55 meV, is much higher than graphene [12]. In silicene, FET requires a large supply voltage of about 30 V to turn on and a band gap drops it down to 0.1 eV [13]. The sensing material of silicene provides a higher efficiency and lower carrier density due to a strong silicene trail for high performance FETs, large band gap, and high Fermi velocity ($5.2\times 10^{5}$ m-S) [14]. These advantages made silicene an endurable fluidity for chemical and biological sensing that can be mobilized with nano electronics technology [15]. In addition to field effect transistor, silicene opens new opportunities due to its tunable band gap whereas graphene indicates zero band gap. It can be stated that silicene is much more reliable as well as much stronger than graphene [16]. In contrast to graphene, silicene has sp3 hybridization instead of sp2 hybridization, which is conducive to potential interaction including exterior particles. For active conduction of atomic sensing, silicene is a promising material used in molecular detection techniques [17].

Silicene, with a “buckling atomic structure”, has double surfaces that are comparable to nanocarbon materials in terms of high area to volume ratio. The addition of vacancy defects to silicene doping improves the quantum capacitance of silicene-based electrodes [18]. The improvement of quantum capacitance is found due to the presence of localized states on every side of the Fermi stage.

Due to the advantages mentioned above, silicene with buckling atomic structure, in contrast to graphene, draws our attention to investigating its use in the detection of label free DNA hybridization. To the best of the authors’ knowledge, the frequency readout of DNA hybridization using silicene’s quantum capacitance has not been investigated yet. The contributions of this paper are outlined below:

- An ion-sensitive silicene-based FETs (ISFETs) structure with an electrolytic solution and a silicene surface layer has been proposed. It has the potential to detect DNA hybridization via quantum capacitance dependent frequency readout.
- An analytical model has been introduced for the measurement of quantum capacitance.
- A comparative study has been performed to find out the most efficient and flexible scheme in silicene quantum capacitance with the help of an LC circuit-based system modeling in contrast to graphene.

The remainder of this paper is outlined as follows. Section 2 introduces design principle and the analytical method of silicene and graphene quantum capacitance. The system model of DNA hybridization detection and pH detection are described in Section 3. The performance of silicene and graphene quantum capacitance in terms of frequency readout is presented in Section 4. The concluding remarks are provided in Section 5.

## 2. Design Principle

### 2.1. ISFET Structure with Silicene and Electrolyte

Recently, charge-detection biosensors are the primary concern of biosensor research, particularly FETs which incorporate high-input impedance, low output impedance compact-structure, and are inexpensive, to get simple and stable “in vivo diagnostic systems“. Nevertheless, the detection of charge from DNA hybridization using ISFETs has recently been used. The metal oxide-semiconductor (MOS) structure of the FET-based device induces bending in the energy band of the semiconductor channel, which also changes the carrier concentration. In MOSFET, we purposefully add the gate potential to switch on the transistor. Any changes in biomolecule or solution potentials (pH value) also affect the gate potential (that is, source-drain voltage/current changes) of the FET-based devices. Such a characteristic change is reflected in the change in the threshold voltage ($V_{th}$).

**Figure 1:**
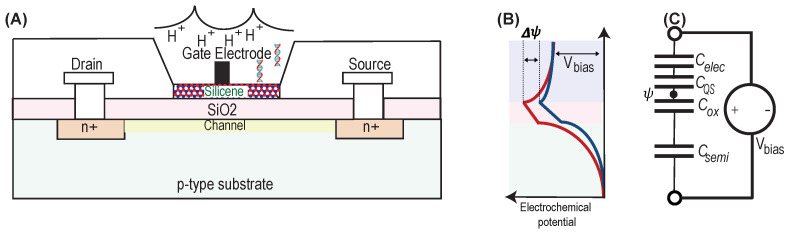
Design principle: (**A**) A simplified model of ISFET with silicene and the electrolyte, (**B**) electropotential curve of ISFET, and (**C**) equivalent capacitor model.

When a match between a DNA target and a DNA probe occurs, the hydrogen particles (H+) are released, which also changes the pH of a fluid. Since the ISFET is specific to H+, it can detect changes in pH as a result of DNA detection using the DNA chain extension law. As shown in Figure 1, an ISFET was first realized by replacing the gate metal with a remote gate, also called the standard fluid junction reference electrode, to set a steady potential or gate bias to the fluid bearing a chemical-sensitive membrane insulator to the electrolyte. The ions in the fluid influence the behavior of H+ and the insulating layer which is used to contain protons on its surface. The pH can be calculated by pH=log([H+]). The changes in the ionic concentration can result in changes in the ISFET channel charge diffusion which eventually changes the threshold voltage $V_{th}$.

Figure 1A demonstrates the ISFET structure that is identical to the MOS model when the electrolytic solution and a silicene surface layer included as a chemical-sensitive membrane are used in the substitution for metal. As the differential surface potentials (Δψ) change in the charge carrier concentration of the channel, the potential difference is developed because of change in surface charge concentration, dσ. It can be related to intrinsic charge from biomolecules/ions emitted during enzymatic reactions. Once the solid–liquid interface model is formed using silicene surface, the ions are divided into three layers because of the local coulomb force. These layers are stern, diffuse, and bulk solution layers. Stern layer: The concentration of ions in this layer is determined by the charges in the solid, and these ions are tightly bound to the surface. As a result, the electric potential is higher at the interface and decreases as one moves closer to the solution. Diffuse layer: The ion density in the diffuse layer changes with the distance from the solid-interface which follows Maxwell–Boltzmann statistics. Bulk solution layers: This layer has less coulomb electrostatic force, and the electric field delays exponentially from the diffusion layer to the bulk solution. The Debye length is the distance between the solid–liquid interface and the boundary where the electric field drops at the rate of $e^{-1}$. In the Debye length, the analyte charges can influence surface characteristics ( Figure 1B).

These three layers can be represented by an analogous capacitor model because of the presence of ions in these layers. Figure 1C shows a capacitor model that consists of four capacitors. The FET capacitance ($C_{F}$) is a series combination of $C_{semi}$, and $C_{OX}$ which are due to the presence of a semiconductor layer (substrate) and dielectric (oxide) layer, respectively. The double layer capacitor ($C_{DL}$) is the series combination of two capacitors $C_{elec}$ and $C_{QS}$, which are due to the capacitance effect of the stern and diffuse layers, respectively. The $C_{QS}$ is also called quantum capacitance. The total capacitance of the ISFET ($C_{ISFET}$) is the parallel combination of $C_{F}$ and $C_{DL}$. The $C_{DL}$ is written as an Equation (1)

(1) $$\frac{1}{C_{DL}}=\frac{1}{C_{elec}}+\frac{1}{C_{QS}};$$

The $C_{F}$ can be expressed as Equation (2)

(2) $$\frac{1}{C_{F}}=\frac{1}{C_{semi}}+\frac{1}{C_{OX}};$$

and the $C_{ISFET}$ can be expressed as Equation (3)

(3) $$C_{ISFET}=C_{F}+C_{DL}$$

The electrochemical potential ψ due to chemical reaction of the surface can be given as in Equation (4) [19].

(4) $$\psi =\frac{d\sigma }{C_{ISFET}}.$$

Since $C_{OX}>>C_{semi}$ and $C_{elec}>>C_{QS}$, thus, using Equations (1)–(3), we can say that $C_{ISFET}$ is dominated by the quantum capacitance of silicene, $C_{QS}$.

The equation for the drain to source current ($I_{DS}$) of an ISFET can be expressed as Equation (5).

(5) $$I_{DS}=\frac{\mu C_{OX}W}{L}\left[\left(V_{GS}-V_{th}\right)V_{DS}-\frac{V_{DS}^{2}}{2}\right],$$

where μ is the electron mobility, *W* is the channel width, *L* is the channel length, $V_{GS}$ is the Gate–Source voltage, $V_{DS}$ is the Drain–Source voltage. The oxide capacitance $C_{OX}$ is expressed as Equation (6)

(6) $$C_{OX}=3.9\times \frac{\epsilon_{0}}{EOT}\left(L\times W\times N\right),$$

where $\epsilon_{0}$ is the permittivity of free space, *N* is the number of gate fingers, and EOT denotes the equivalent oxide thickness.

The threshold voltage can be expressed as Equation (7)

(7) $$V_{th}=E_{ref}-\psi +\chi_{sol}-\phi_{si}-\frac{Q_{ox}+Q_{ss}+Q_{B}}{C_{OX}}+2\phi_{F},$$

where $E_{ref}$ is the potential difference between liquid and reference electrode of the surface, $\chi_{sol}$ is the surface dipole potential of the solution, $\phi_{si}$ is the work function of bulk semiconductor, $Q_{ox}$ is the accumulated charge in the oxide, $Q_{ss}$ is the fixed surface-state charge per unit area at the insulator semiconductor interface, $Q_{B}$ is the semiconductor depletion charge per unit area, and $\phi_{F}$ is the Fermi potential of the semiconductor.

The overall sensitivity (denoted by $\frac{dI_{DS}}{I_{DS}}$) of the FET-based biosensor consists of three states—Stage 1: the charge concentration changes near the sensor interface as the concentration of analytes changes, which is denoted by dσ. Stage 2: The difference in effective gate voltage $dV_{gate}$ is caused by a change in charge concentration. Stage 3: The drain current shift (i.e., $dI_{DS}$) is caused by the change in $V_{gate}$, which can be determined using the current–voltage characteristics curve.

(8) $$\frac{dI_{DS}}{I_{DS}}=dn\times \frac{d\sigma }{dn}\times \frac{dV_{gate}}{d\sigma }\times \frac{dI_{DS}/dV_{gate}}{I_{0}},$$

where *n* is the concentration of the hydrogen ions (or carrier concentration).

### 2.2. Quantum Capacitance Model of Silicene and Graphene

An LC system is based on a resonator circuit, which consists of an inductor and a capacitor that operate as resonant frequency. While using silicene, the quantum capacitance-based wireless sensor is connected to an inductor, and thus the resonant frequency changes in accordance with quantum capacity. On the other hand, with DNA hybridization, the quantum capacitance is changed. The LC circuit can, therefore, be used as a DNA sensor.

The quantum capacitance of silicene ($C_{QS}$) can be expressed by Equation (9) [20].

(9) $$C_{QS}=\frac{2e^{2}kT}{\pi {\left(h\nu_{F}\right)}^{2}}\times \ln\left[2\left(1+\cosh\frac{E_{F}}{kT}\right)\right]\times \left(L\times W\times N\right),$$

where $E_{F}$ is the Fermi-energy level, *e* is the electron’s charge, *T* is the temperature in Kelvin, $\nu_{F}$ is the Fermi velocity, *h* and *k* are Planck’s and Boltzmann constants, respectively.

If $E_{F}>>kT$, then Equation (10) can be modified as below

(10) $$C_{QS}=\frac{2e^{2}}{\left(h\nu_{F}\right)\sqrt{\pi }}\sqrt{n}\left(L\times W\times N\right),$$

where *n* is the net carrier concentration. In a silicene device, carrier concentration can be defined as $n=|n_{G}\left|+\right|n^{*}|$, where $n_{G}$ is the carrier concentration $n_{G}={\left(\frac{eV}{h\nu_{F}\sqrt{\pi }}\right)}^{2}$ and $n^{*}$ is the external charge on the silicene surface.

To introduce a conceptual prognosis of quantum capacitance $C_{QG}$ for ideal graphene, it can be expressed [21] as the form of Equation (11)

(11) $$C_{QG}=\frac{2e^{2}kT}{\pi {\left(h\nu_{F}\right)}^{2}}\times \ln\left[2\left(1+\cosh\frac{eV_{ch}}{kT}\right)\right],$$

where the Fermi velocity of the Dirac electron $\nu_{F}=c/300$ and $V_{ch}=E_{F}/e$ are the potential of graphene.

(12) $$C_{QG}\approx e^{2}\frac{2eV_{ch}}{\pi {\left(h\nu_{F}\right)}^{2}}=\frac{2eV_{ch}}{\sqrt{\pi }{\left(h\nu_{F}\right)}^{2}}\sqrt{n}.$$

The resonant frequency of L and $C_{QJ}$ (where J=S for silicene and J=G for graphene) of the device can be expressed as in Equation (13).

(13) $$f=\frac{1}{2\pi \sqrt{LC_{QJ}}},$$

**Figure 2:**
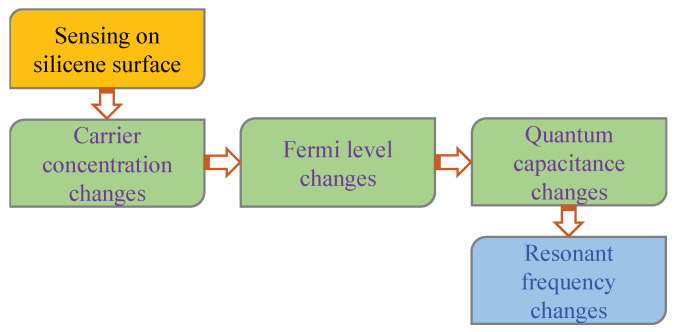
Transduction method of silicene quantum capacitance-based frequency readout.

Figure 2 shows a functional block diagram for silicene quantum capacitance based on frequency readout. When a DNA sample interacts with a buffer solution (such as phosphate buffer (PB), and phosphate buffer saline (PBS)), the carrier concentration (H+) on the silicene surface changes, which affects Fermi velocity and quantum capacitance. Then, this quantum capacitance changes the resonant frequency of the LC circuit.

## 3. System Model Based on Detection Principle

### 3.1. DNA Hybridization Process

**Figure 3:**
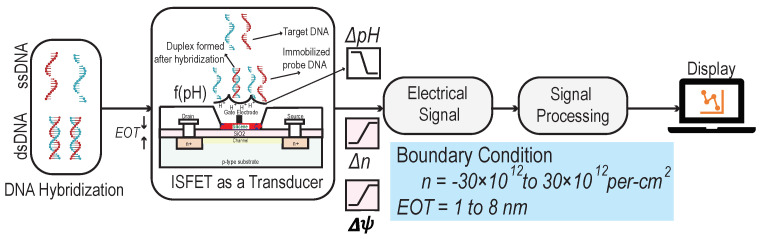
Schematic and block diagrammatic representation of the generation of electrical signal from the DNA hybridization.

By DNA–DNA hybridization, the DNA probes, in the sections of ssDNA, detect the existence of complementary nucleic acid sequences (G-T-A-C). The ISFET with a two-dimensional channel made of a silicene layer can be worked as a field effect charge sensor or pH sensor (see Figure 1). Such a sensor can be used for the label-free detection of DNA hybridization. The ISFET with an aqueous solution (such as PBS) and silicene layer detects intrinsic charges or charge transfer during chemical reactions, which is used to quantify DNA hybridization. The electrical detection of single-strand probe DNA (ssDNA) and double-strand probe DNA (dsDNA) is made possible when DNA hybridization forms non-covalent hydrogen bonds in PBS solutions by contributing ions on the silicene layer which indeed changes the electrical properties of the ISFET device [22]. The quantum capacitance at the electrolyte–solid interface depends on the covalent immobilization of the ssDNA probe on the solid surface. The extracted electrical signal from the ISFET device is then amplified and processed for the readout or display. This process is shown in Figure 3.

### 3.2. pH Detection Model

**Figure 4:**
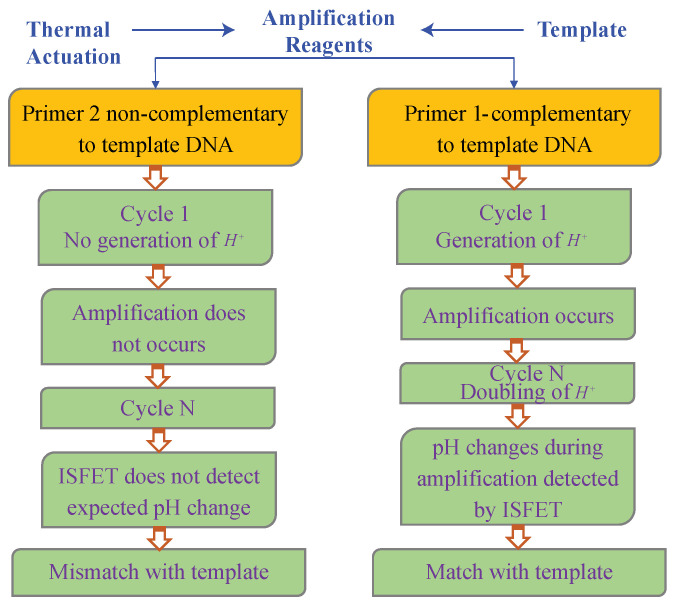
pH detection model.

Fast and dependable detection is the most common application of DNA biosensors, therefore, new DNA hybridization biosensors have received much attention from researchers [23]. However, this practice typically includes more complicated sample preparations and measuring tools to achieve more robust sensing. The samples consist of many prime target analysts that can detect DNA hybridization. Several targets in one reaction are particularly essential for multiplexing DNA hybridization detection. The operation process is acquired by integrating multiple transistors into a single array of different DNA samples [24]. A label-free detection is calmed, and hybridization can be done at a low pH salt level based on the hybridization of DNA samples using the electrostatic repulsion between the sample and destination. The ISFET is used for solution measurements of ion concentrations. When the ion concentrations (H+) change, pH levels will change accordingly; as a result, the current is changed through the transistor [25]. The ion concentration solution is used here as the gate electrode. Figure 4 shows a label-free real-time pH detection model analysis.

The chemical reaction on the surface of silicene changes the charge density on the channel by applying the gate voltage. The induced charge density in the FET channel would therefore be changed. As a result, the drain to source current $I_{DS}$ is modified corresponding to pH at the SiO${}_{2}$ interface for sensing on silicene. The complementary and non-complementary tests are conducted with a DNA sequencing template, amplification reagents, and thermal actuation combinations. In the complementary DNA template, H+ ions are formed, and double H+ ions are amplified. The ISFET changes pH, and amplification is detected that is matched with the template. Therefore, pH is detected with DNA hybridization. On the other hand, the non-complementary DNA template does not produce H+, and no amplification occurs; that is, the cycle does not hybridize. Therefore, the ISFET does not detect the expected pH change, which is a mismatch with sequencing reagents.

**Figure 5:**
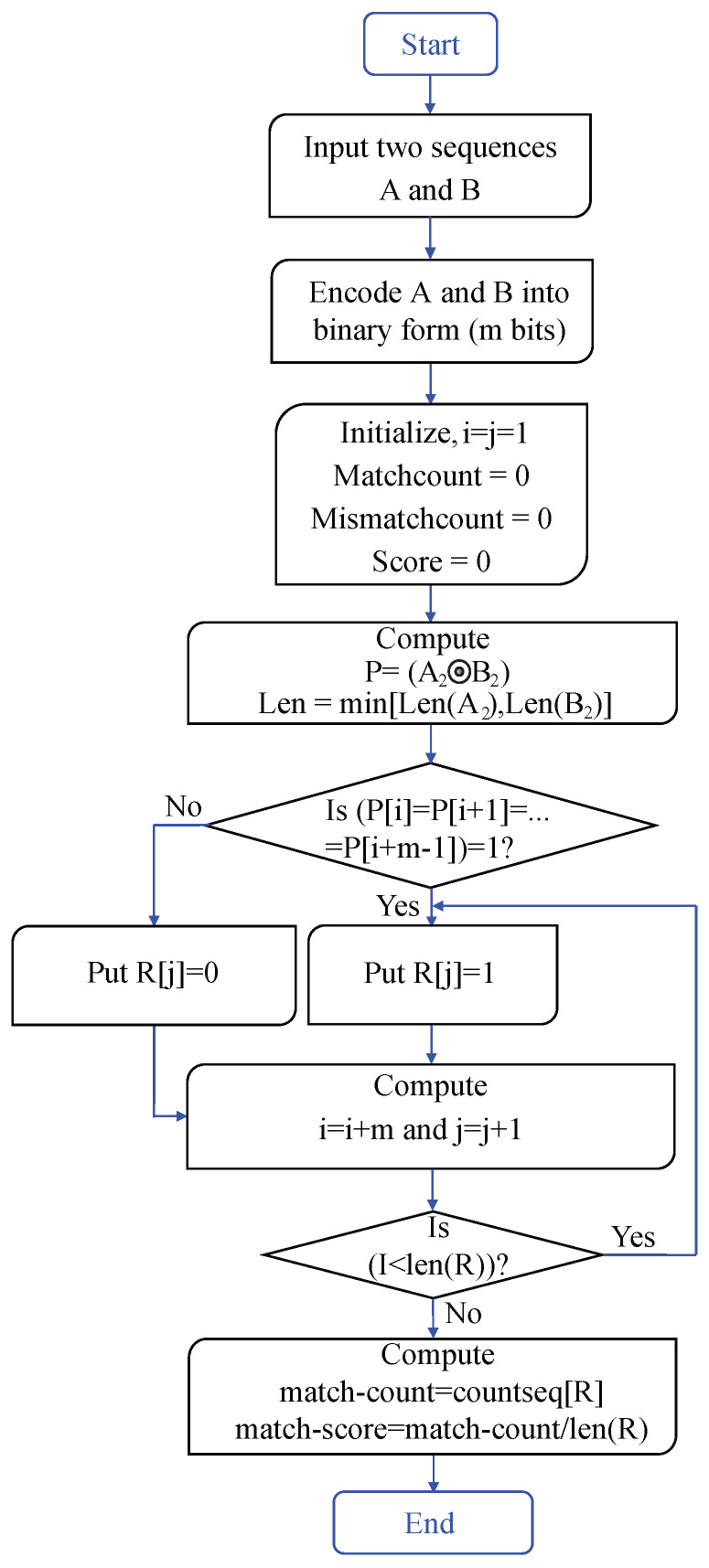
Flowchart of DNA hybridization in two samples A and B.

The algorithm in Figure 5 finds the percentage of similarity (Match-Score) of the two biological sequences A and B. The two input sequences A and B are ssDNA that report on complementary DNA or mismatched DNA are called target probe DNA. The complementary DNA produce the results of entirely matched dsDNA and mismatched DNA. The Boolean algebra is used to calculate the Match-Score. In this algorithm, firstly, two DNA sequences are encoded into binary numbers. Secondly, compute the similarity and minimum length of these binary encoded sequences. Finally, the match count and Match-Score are calculated.

## 4. Experimental Analysis and Results

### 4.1. Experiment Setup

This section compares the performance of silicene and graphene in terms of frequency readout. This analysis takes into account the average frequency. The quantum capacitance is affected by carrier concentration when DNA hybridization occurs. Figure 3 shows the DNA hybridization process where the hydrogen ions are generated. The process changes the carrier concentration, the electrochemical potential, as well as the quantum capacitance of the ISFET device. The corresponding electrical signal is amplified and pre-processed before being displayed.

**Figure 6:**
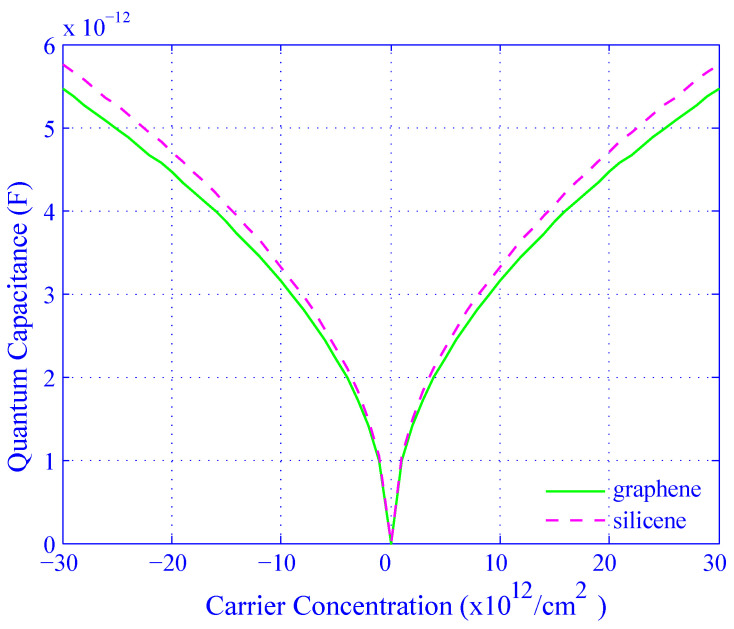
Effect of carrier concentration on quantum capacitance for both silicene (dotted line) and graphene (solid line).

In this paper, we compared silicene and graphene in terms of $C_{QJ}$ where J∈{S,G} as a function of *n* (see Figure 6); $C_{OX}$ as a function of EOT; and quantum frequency as a function of $C_{QJ}$ as well as *n*. For the numerical simulation, we varied the value of *n* from $-30\times 10^{12}$ to $30\times 10^{12}$ per cm${}^{2}$ and EOT from 1 to 8 nm [3,26].

The silicene (also graphene) channel of the scheme is associated with the inductor that invents a resonant circuit. The $C_{QJ}$ plays a role in changing the resonant frequency of the devices (see Equation (13)). With the use of MATLAB tools, simulation has been conducted and the results are presented for an ideal channel without any distortion. The simulation was carried out by considering the parameters shown in Table 1 for ideal silicene and graphene channels of the devices, respectively.

### 4.2. Performance Metrics

The simulated results for all the schemes of silicene are represented by dotted lines with markers, while that of the graphene are marked by solid lines. The Equations (10) and (12) exhibit the familiarity within quantum capacitance and carrier concentration of silicene and graphene channels of the devices, respectively, and their performances are shown in Figure 6. For the case of graphene, the modulus of the carrier concentration varies from 0 to $30\times 10^{12}/$cm${}^{2}$ and the corresponding quantum capacitance decreases from 5.5 pF to 0 for the negative carrier concentration and it increases from 0 to 5.5 pF for the positive carrier concentration. As well as for silicene, the behavior of the quantum capacitance variation with carrier concentration is similar to that of graphene except the peak value of quantum capacitance is 5.8 pF which is much sharper than graphene. When we measured the quantum capacitance versus carrier concentration of label-free DNA hybridization, we got quantum capacitance up to 5.8 pF for silicene which was remarkable. It may conclude that carrier concentration is more sensitive with silicene quantum capacitance than graphene.

**Figure 7:**
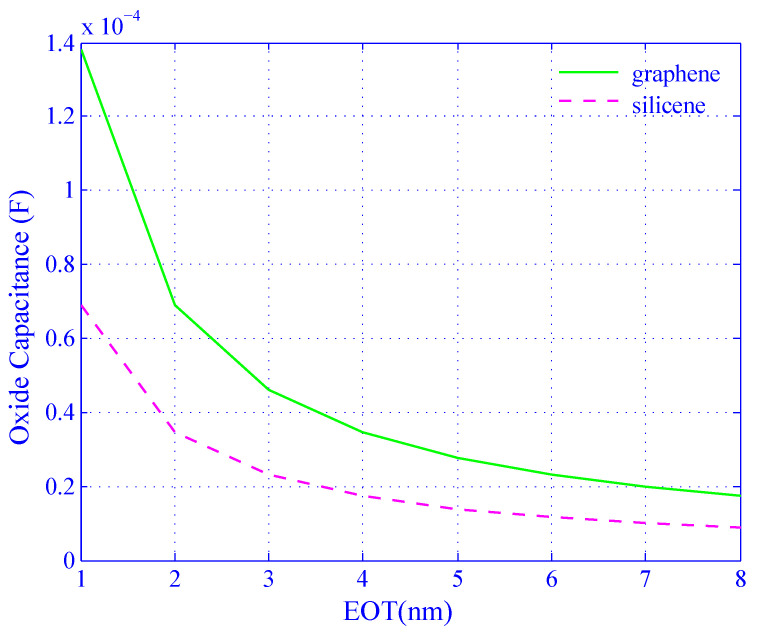
Oxide capacitance decreases with effective oxide thickness (EOT) (nm) for both silicene (dotted line) and graphene (solid line).

Figure 7 shows the oxide capacitance as a function of effective oxide thickness (EOT) which is a relay on the area of gate oxide thickness. The channel of the device capacitance depends on the EOT when the device gate area is fixed. From the results, it is found that with the increasing of the EOT the oxide capacitance decreases exponentially for both the cases of silicene and graphene. With the variation of the EOT from 1 nm to 8 nm, the oxide capacitance decreases for both graphene and silicene from 14 mF to 1.9 mF and 6.5 mF to 1.5 mF, respectively, while the quantum capacitance was kept maximum for both cases.

**Figure 8:**
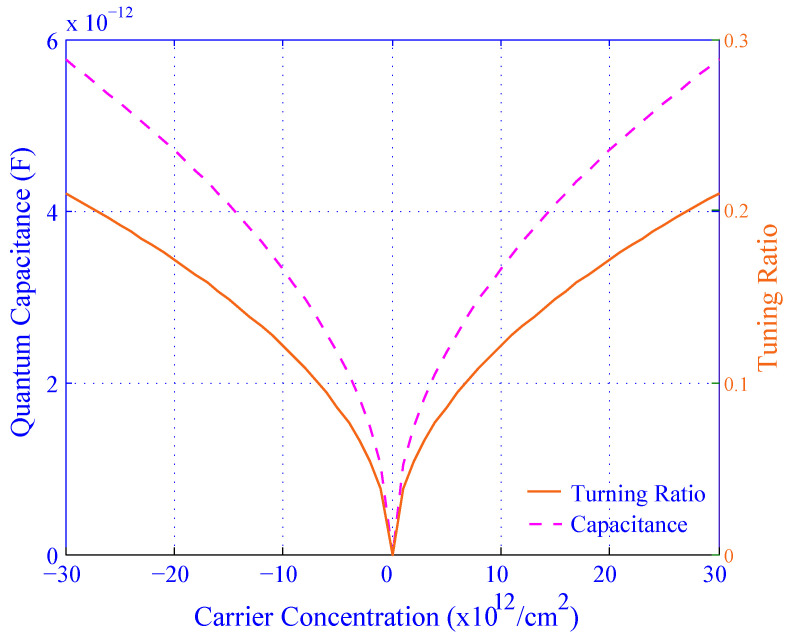
The effect of carrier concentration on the quantum capacitance and tuning ratio for silicene.

Figure 8 represents the quantum capacitance and tuning ratio as a function of carrier concentration for silicene. The graph is divided into halves on the X-axis to explain the characteristics of the graph properly. The first and second halves are considered as a function of the carrier concentration from $-30\times 10^{12}$/cm${}^{2}$ to 0 and 0 to $30\times 10^{12}$/cm${}^{2}$, respectively. Both the capacitance and tuning ratio decrease sharply for the negative carrier concentration (the first half) whereas the capacitance and tuning ratio increase rapidly for the positive carrier concentration (the second half). The peak value of the capacitance and tuning ratio of the channel are 5.8 pF and 0.2, respectively.

**Figure 9:**
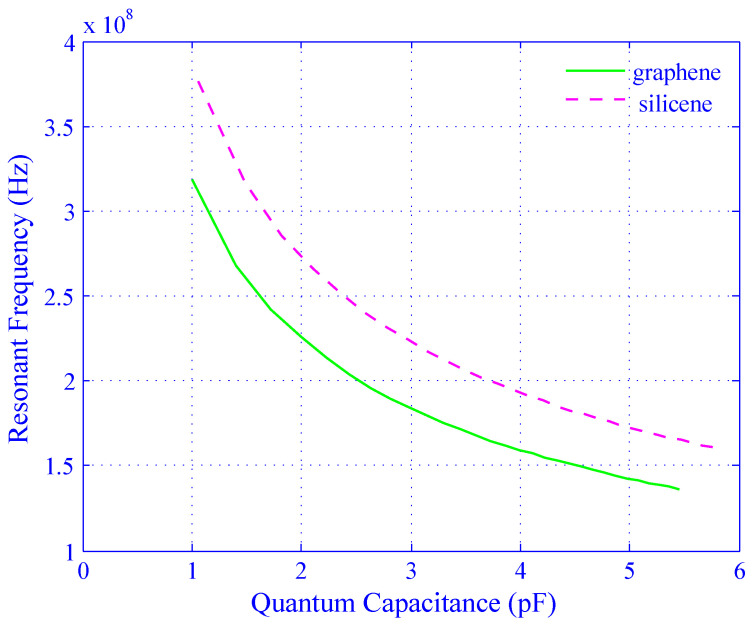
Effect of the quantum capacitance ($\times 10^{-12}$ F) on resonant frequency for both the silicene (dotted line) and graphene (solid line).

The resonant frequency depends on the quantum capacitance and the inductance. At zero gate voltage, the value of the inductor, L≈250 nH, was ascertained to conduct a frequency f=1 GHz. Figure 9 shows the effect of quantum capacitance of the silicene and graphene on their respective resonant frequencies for the fixed value of L. When the values of quantum capacitance change from 1 pF to 6 pF, the resonant frequencies of both silicene and graphene decrease exponentially. The peak values of resonant frequency are found to be $3.8\times 10^{8}$ Hz and $3.3\times 10^{8}$ Hz (using Equations (10), (12), and (13)) for silicene and graphene, respectively.

**Figure 10:**
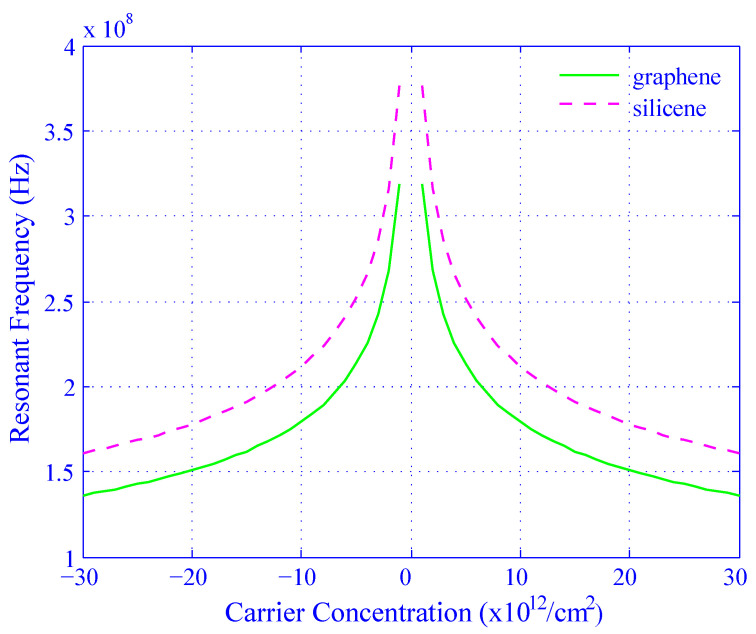
Effect of carrier concentration on the resonant frequency for both silicene (dotted line) and graphene (solid line).

Figure 10 illustrates the effect on carrier concentration on the quantum capacitance and resonant frequency for silicene and graphene. The resonant frequency has achieved L=250 nH using Equation (13) discussed in Section 2.2. The graph is divided into halves on the X-axis to explain the characteristics properly. The first and second halves are considered as a function of the carrier concentration from $-30\times 10^{12}$/cm${}^{2}$ to 0 and 0 to $30\times 10^{12}$/cm${}^{2}$, respectively. For both cases, the frequency increases very rapidly for the negative carrier concentration (the first half) whereas the frequency decreases very sharply for the positive carrier concentration (the second half). The minimum and maximum frequencies are $1.6\times 10^{8}$ Hz and $3.8\times 10^{8}$ Hz, respectively, for silicene and the values are $1.4\times 10^{8}$ Hz and $3.2\times 10^{8}$ Hz, respectively, for graphene. It is observed from Figure 9 that the change of resonant frequency with respect to the carrier concentration for silicene is sharper than that of graphene. It is also found that the peak amplitude of the resonant frequency for silicene is higher than that of graphene. The observation shows that (Figure 9 and Figure 10) silicene is more sensitive than graphene as a sensing material.

**Figure 11:**
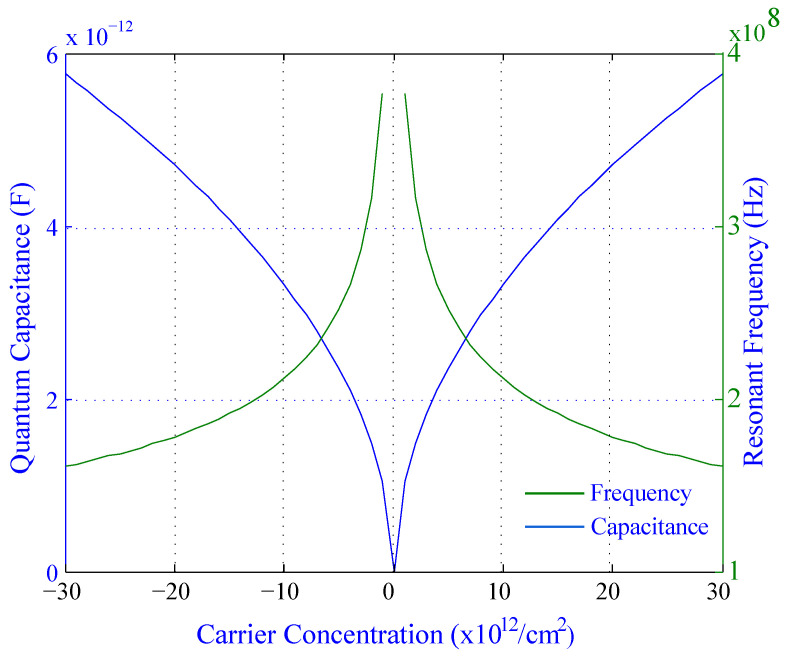
Effect of carrier concentration on the quantum capacitance as well as resonant frequency for silicene.

Figure 11 shows the effect of carrier concentration on the quantum capacitance and resonant frequency for silicene. The graph can be divided into halves on the X-axis to explain the characteristics of the graph properly. The first and second halves depends on the carrier concentration from $-30\times 10^{12}$/cm${}^{2}$ to 0 and 0 to $30\times 10^{12}$/cm${}^{2}$, respectively. The Y-axis (Left) represents the values of quantum capacitance within the range from 0 to $6\times 10^{-12}$ F and the Y-axis (Right) represents the resonant frequency within the range from $1\times 10^{8}$ to $4\times 10^{8}$ Hz. In negative carrier concentration (the first half), the quantum capacitance decreases whereas the resonant frequency increases. On the other hand, in a positive carrier concentration (the second half), the quantum capacitance increases when the resonant frequency decreases. Therefore, the frequency and capacitance are inversely proportional to carrier concentration and the peak value of the quantum capacitance and the lowest value of resonant frequency are $5.8\times 10^{-12}$ F and $1.6\times 10^{8}$ Hz, respectively.

From Figure 8 to Figure 11, the changes in quantum capacitance, resonant frequency, and tuning ratio indicate that the sensitivity of silicene is much more effective than graphene.

## 5. Conclusions

The 2D material silicene is used as a highly sensitive molecular sensor that has lower charge density than graphene. Silicene has a band gap which makes it suitable to be used in novel transistors and others. An ISFET structure with electrolytic solution and a silicene surface layer has been proposed. It has the potential to detect DNA hybridization via quantum capacitance dependent frequency readout. The comparison between silicene and graphene was performed based on the LC circuit modeling and via the resonant frequency of the system. In the presence of a silicene channel device, it is shown that when the carrier concentration is increased, the capacitance is also sharply increased compared to graphene. The frequency response is nearly linear in regards to the DNA concentration. Therefore, to continue the operating performance, the device for material sensing of silicene is added to label-free detection of DNA hybridization which demonstrated the frequency readout of the systems. The greatest hurdles for meeting demands for point-of-care diagnostics are the sample preparation, reagent handling, and the fact that it cannot be used in low resource settings. Thus, further investigation is required for enzyme or nanoparticles label-based electrochemical detection and microfluidic technology, which incorporates nucleic acid extraction, amplification, and detection on a single device.

## Figures and Tables

**Table 1:** Simulation parameters.

Parameter	Value	Unit
Properties of Silicene [9,27,28]		
Band gap	0.1 –0.5	eV
Intrinsic mobility	1000	cm${}^{2}$/V.s
Fermi velocity	$5.21\times 10^{5}$	ms${}^{-1}$
Oxide thickness	6	$\dot{A}$
Residual carrier density	$10^{-9}$ –$10^{-12}$	cm${}^{-2}$
Field effect mobility	100 –200	cm${}^{2}$/V.s
Gate length	1	m
Properties of Graphene [29,30]		
Band gap	0	eV
Intrinsic mobility	4000	cm${}^{2}$/V.s
Fermi velocity	$1.1\times 10^{6}$	ms${}^{-1}$
Oxide thickness	5	$\dot{A}$
Residual carrier density	$10^{-10}$ –$10^{-12}$	cm${}^{-2}$
Field effect mobility	1800	cm${}^{2}$/V.s
Gate length	0.2	m
Other Properties		
No. of finger	200	
Finger width	10	m
Temperature	300	k
Plank’s constant	$6.6\times 10^{-34}$	m${}^{2}$ kg/s
Dielectric constant	$8.854\times 10^{12}$	f/m
Charge of electron	$1.6\times 10^{-19}$	Coulomb
Boltzmann constant	$1.3\times 10^{-23}$	m${}^{2}$ kg s${}^{-2}$ K${}^{-1}$

## Data Availability

Not applicable.

## Abbreviations

The following abbreviations and symbols are used in this manuscript:

2D	Two Dimensional
cDNA	Complementary DNA
dsDNA	Double-strand DNA
ssDNA	Single-strand-DNA
DNA	Deoxyribonucleic Acid
EOT	Effective Oxide Thickness
FET	Field Effect Transistor
GNR	Graphene Nano-ribbon
ISFET	Ion-sensitive FET
LC	Inductor and Capacitor Circuit
MOS	Metal Oxide Semiconductor
MOSFET	Metal Oxide Semiconductor Field Effect Transistor
PB	Phosphate Buffer
PBS	Phosphate Buffer Saline
SiNR	Silicene Nano-ribbon
SiNW	Silicene Nanowires
$C_{G}$	Gate Capacitance
$C_{DL}$	Double Layer Capacitance
$C_{F}$	FET Capacitance
$C_{OX}$	Oxide Capacitance
$C_{Q}$	Quantum Capacitance
$C_{Q_{G}}$	Quantum Capacitance of Graphene
$C_{Q_{S}}$	Quantum Capacitance of Silicene
